# Flagellar pocket restructuring through the *Leishmania* life cycle involves a discrete flagellum attachment zone

**DOI:** 10.1242/jcs.183152

**Published:** 2016-02-15

**Authors:** Richard J. Wheeler, Jack D. Sunter, Keith Gull

**Affiliations:** 1 Max Planck Institute of Molecular Cell Biology and Genetics, Pfotenhauerstraße 108, Dresden 01307, Germany; 2 Sir William Dunn School of Pathology, South Parks Road, Oxford OX1 3RE, UK

**Keywords:** *Leishmania*, *Trypanosoma brucei*, Amastigote, Electron tomography, Flagellum attachment zone, Life cycle differentiation

## Abstract

*Leishmania* promastigote parasites have a flagellum, which protrudes from the flagellar pocket at the cell anterior, yet, surprisingly, have homologs of many flagellum attachment zone (FAZ) proteins – proteins used in the related *Trypanosoma* species to laterally attach the flagellum to the cell body from the flagellar pocket to the cell posterior. Here, we use seven *Leishmania mexicana* cell lines that expressed eYFP fusions of FAZ protein homologs to show that the *Leishmania* flagellar pocket includes a FAZ structure. Electron tomography revealed a precisely defined 3D organisation for both the flagellar pocket and FAZ, with striking similarities to those of *Trypanosoma brucei*. Expression of two *T. brucei* FAZ proteins in *L. mexicana* showed that *T. brucei* FAZ proteins can assemble into the *Leishmania* FAZ structure. *Leishmania* therefore have a previously unrecognised FAZ structure, which we show undergoes major structural reorganisation in the transition from the promastigote (sandfly vector) to amastigote (in mammalian macrophages). Morphogenesis of the *Leishmania* flagellar pocket, a structure important for pathogenicity, is therefore intimately associated with a FAZ; a finding with implications for understanding shape changes involving component modules during evolution.

## Abstract (precis)

**Summary:** *Leishmania* parasites have a highly structured flagellar pocket, including a structure homologous to the *Trypanosoma brucei* flagellum attachment zone, which undergoes structural adaptations in different life cycle stages.

## INTRODUCTION

*Trypanosoma* and *Leishmania* are two related genera that include many major human and livestock pathogens, such as *Leishmania mexicana*, which causes New World cutaneous leishmaniasis, and *Trypanosoma brucei*, which causes African sleeping sickness. Like many unicellular eukaryotes, these cells have a highly defined internal organisation (Lacomble et al., 2009, 2010; Sherwin and Gull, 1989), and undergo precise morphogenesis during division (Ambit et al., 2011; Robinson et al., 1995; Sheriff et al., 2014; Sherwin and Gull, 1989; Wheeler et al., 2011, 2013a) and precise morphogenesis during adaptation of cell shape to different environments (Gadelha et al., 2013; Rotureau et al., 2011, 2012; Sharma et al., 2008; Wheeler et al., 2015). How they generate their shape is of interest because of its apparent co-evolution with different pathogenic life cycles and the capacity of parasites to persist in different niches within their hosts (Maslov et al., 2013; Wheeler et al., 2013b).

Trypanosomes and *Leishmania* most likely arose from an ancestor with a promastigote *Leishmania*-like morphology – an ovoid cell with a single flagellum protruding from the flagellar pocket at the anterior (Flegontov et al., 2013). The flagellar pocket is central to the morphogenesis of trypanosomes and is associated with many single copy organelles – the basal body and pro-basal body pair, the flagellum, the Golgi and the mitochondrial DNA (the kinetoplast) (Gheiratmand et al., 2013; Gull, 2003; Lacomble et al., 2009, 2010). The pocket is also the site of all endo- and exocytosis and hence has a crucial role in the pathogenicity of the parasite (Engstler et al., 2007; Field and Carrington, 2009; Gadelha et al., 2009). Correct pocket formation is vital for cell morphogenesis, viability and infectivity, as evidenced in *T. brucei* (Absalon et al., 2008; Bonhivers et al., 2008).

Trypanosomes, uniquely among genera of the Trypanosomatidae family, have developed a life cycle that includes a free-swimming stage in the vertebrate bloodstream. This genus has the innovation of trypomastigote morphology with the flagellar pocket towards the cell posterior and a flagellum that is laterally attached to the cell surface and runs towards the anterior. The lateral attachment of the flagellum is mediated by a large complex cytoskeletal structure called the flagellum attachment zone (FAZ), which connects the flagellar skeleton to the cell body cytoskeleton through both the flagellum and cell body membranes (Hayes et al., 2014; Höög et al., 2012; Robinson et al., 1995; Sherwin and Gull, 1989; Vaughan et al., 2008; Vickerman, 1969). The morphological innovation of the FAZ seems to be associated with adaptation to the host environment of the bloodstream (Wheeler et al., 2013b). Flagellar pocket and FAZ formation are linked; the proximal end of the FAZ (in the flagellar pocket) is the site of FAZ assembly (Sunter et al., 2015; Zhou et al., 2015), and the FAZ is physically connected with the flagellar pocket cytoskeleton through microtubules (Lacomble et al., 2009, 2010) and the bi-lobe structure (Esson et al., 2012; Gheiratmand et al., 2013). In *T. brucei*, correct FAZ formation is vital for cell morphogenesis and viability (Hayes et al., 2014; LaCount et al., 2002; Rotureau et al., 2014; Sun et al., 2013; Vaughan et al., 2008; Zhou et al., 2011, 2015).

The flagellar pocket is likely to be important to a similar degree in *Leishmania* species. Evidence for a complex *Leishmania* pocket structure incorporating cytoskeletal components exists from many years of electron microscopy analysis – *Leishmania* have a microtubule quartet next to the flagellar pocket (Molyneux et al., 1975; Weise et al., 2000), at least one specialised cytoplasmic microtubule starting near the pocket (Weise et al., 2000) and electron-dense structures linking the cell body and flagellum (Aleman, 1969; Gadelha et al., 2013; Molyneux et al., 1975). However, little is known about the three-dimensional (3D) organisation of the pocket. *Leishmania* genomes encode homologs of most FAZ proteins (Sunter et al., 2015) but do not have the laterally attached flagellum characteristic of the trypanosome trypomastigote morphology, leaving their function completely unknown. It has been suggested that structures in the *Leishmania* flagellar pocket region correspond to a FAZ-related structure (Gadelha et al., 2013), although this has not been proven. Furthermore, unlike trypanosomes, *Leishmania* undergo a large change in flagellum structure through the life cycle, from a 9+2 axoneme to a collapsed 9+0 (9v) axoneme (Alexander, 1978; Gluenz et al., 2010; Wheeler et al., 2015). How a complex pocket organisation like that of *T. brucei* (Lacomble et al., 2009, 2010) can accommodate this flagellum restructuring is also unknown.

We used a combination of electron tomography analysis and eYFP tagging of *L. mexicana* FAZ protein homologs to determine the 3D structure and molecular composition of the *L. mexicana* flagellar pocket region, which showed that it includes a previously unrecognised structure homologous to the *T. brucei* FAZ. This allowed us to determine how the *T. brucei* FAZ structure evolved from the ancestral morphology, infer potential functional groupings of FAZ proteins and analyse how *Leishmania* use FAZ proteins to adapt their flagellar pocket structure through their life cycle. These results highlight the importance of the expression and regulation of the assembly of cohorts of proteins (which we term component modules) in determining different cell shapes and cell form in both the life cycle and evolution.

## RESULTS

### *L. mexicana* FAZ protein homologs localise to structures adjacent to the pocket

**Fig. 1.**
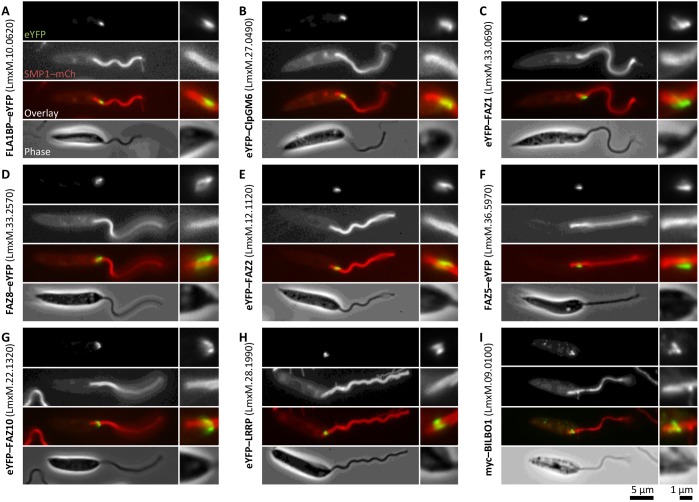
***L. mexicana* FAZ protein homologs localise to the distal flagellar pocket.** Fluorescence micrographs of *L. mexicana* promastigotes expressing the indicated fusion proteins of FAZ and pocket-associated genes from their endogenous loci. SMP1–mCh is a flagellar membrane marker. Detail of the pocket region is shown on the right in each panel. (A–G) Native fluorescence from cells expressing eYFP fusions (eYFP–) of *L. mexicana* homologs of *T. brucei* FAZ proteins. (H) Native fluorescence from a cell expressing an eYFP fusion of the *L. mexicana* homolog of LRRP1, a *T. brucei* bilobe protein. (I) Anti-Myc immunofluorescence of cells expressing the *L. mexicana* homolog of BILBO1, a *T. brucei* pocket collar protein, tagged with the Myc-epitope tag.

There are 34 known FAZ-protein-coding genes in *T. brucei* (Hayes et al., 2014; Hu et al., 2015; LaCount et al., 2002; McAllaster et al., 2015; Morriswood et al., 2013; Nozaki et al., 1996; Oberholzer et al., 2011; Rotureau et al., 2014; Sun et al., 2013; Sunter et al., 2015; Vaughan et al., 2008; Woods et al., 2013; Zhou et al., 2011, 2015), and the majority of these proteins have at least one clear homolog in *L. mexicana* species based on sequence similarity, retention of domains and synteny (Table 1). As *Leishmania* species do not have a flagellum laterally attached to the cell body, the function of this FAZ protein cohort in *Leishmania* is unclear. We therefore generated *L. mexicana* cell lines expressing eYFP fusions of a subset of FAZ homologs to determine their localisation, using the *Leishmania* endogenous tagging plasmid pLEnTv2-YB (Dean et al., 2015). We chose to localise FAZ1 (LmxM.36.5970), FAZ2 (LmxM.12.1130), FAZ5 (LmxM.36.5970), FAZ8 (LmxM33.2570), ClpGM6 (LmxM.27.0490), FLA1BP (LmxM.10.0620) and FAZ10 (LmxM.22.1320) as these proteins are found along the majority of the length of the FAZ in *T. brucei* (accession numbers are given in brackets). All cell lines also expressed mCherry-tagged SMP1 (SMP1–mCh) as a fluorescent marker for the flagellar membrane (Tull et al., 2004; Wheeler et al., 2015), and we confirmed correct integration of the eYFP-tagging constructs and fusion protein expression by using PCR and western blotting for GFP (Fig. S1). All seven fusion proteins localised to the flagellar pocket region in *L. mexicana* promastigotes (Fig. 1A–G), so to further characterise this region of the cell, we also generated cell lines expressing an eYFP fusion of the *L. mexicana* homolog of the bilobe protein LRRP1 (LmxM.28.1990) using pLEnTv2-YB and a Myc-epitope-tag fusion of the flagellar pocket collar protein BILBO1 (LmxM.09.0100) using pPOTv2 and fusion PCR tagging (Dean et al., 2015) as reference structures (Fig. 1H–I). We also attempted immunofluorescence analysis with the L3B2 and L6B3 antibodies against *T. brucei* FAZ1 (Kohl et al., 1999) but saw no signal (data not shown).

**Table 1.**
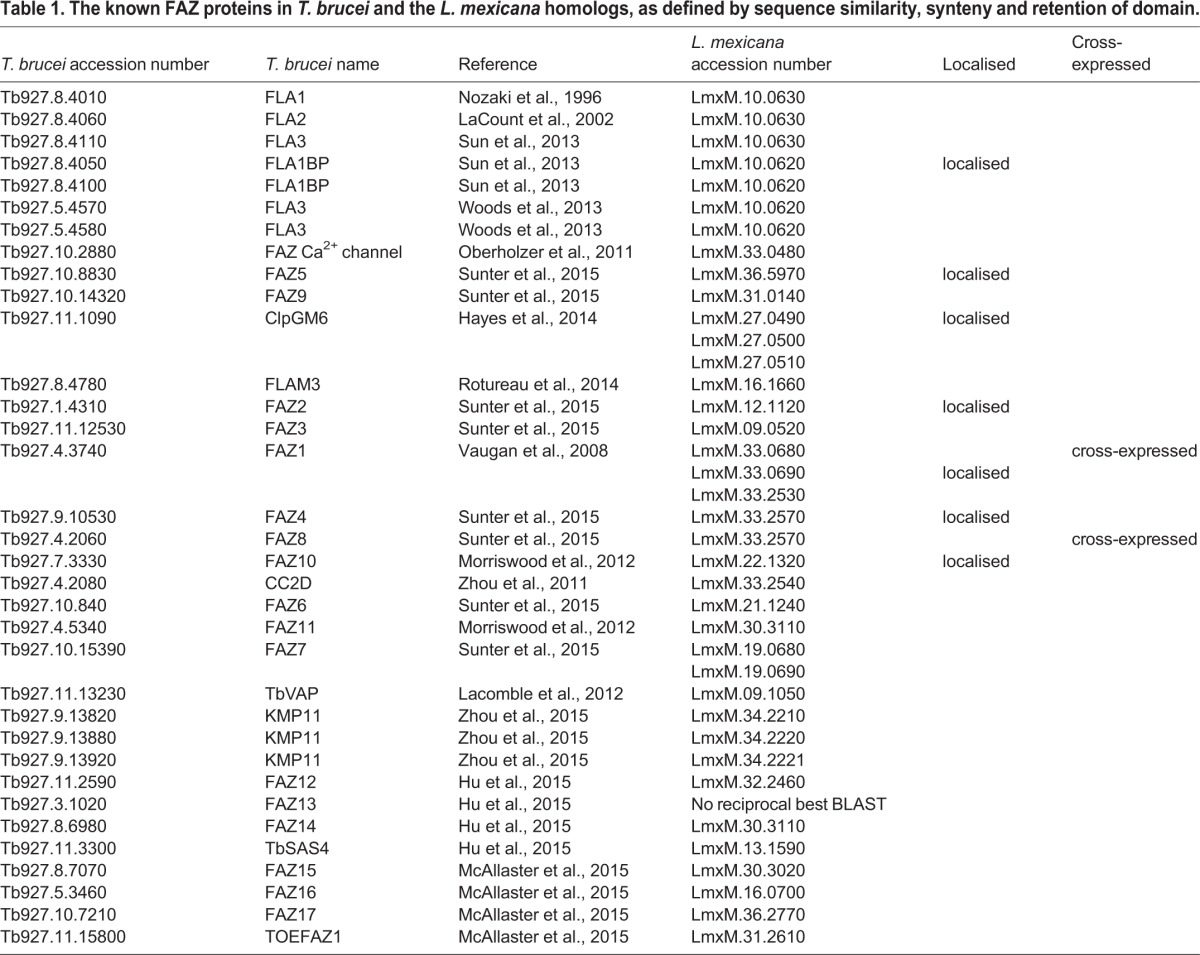
**The known FAZ proteins in *T. brucei* and the *L. mexicana* homologs, as defined by sequence similarity, synteny and retention of domain.**

The flagellar pocket region is identifiable in phase contrast and SMP1–mCh fluorescence images of *Leishmania* cells, where the SMP1–mCh flagellar membrane signal penetrates 1.5–2 μm into the cell anterior. Myc–BILBO1 localised to a line or ring across the flagellum mid-way through the flagellar pocket region, approximately 1 μm from the base of the flagellum (Fig. 1I). All seven eYFP–FAZ protein fusions localised to structures in the pocket region, at either a similar or more distal distance from the base of the flagellum as BILBO1. Localisation of these proteins indicates a complex asymmetric structure because not all proteins had identical localisation patterns and often lay to one side of the flagellum. The localisation patterns could be separated into four classes: a short linear structure either in the flagellum or in very close proximity to the flagellar membrane (ClpGM6, FLA1BP; Fig. 1A,B), a short linear structure to one side of the flagellum (FAZ1, FAZ2, FAZ5, FAZ8; Fig. 1C–F), a ring structure around the flagellum mid-way through the flagellar pocket (FAZ1, FAZ8; Fig. 1C,D), or a horseshoe or ring structure around the exit point of the flagellum from the flagellar pocket (FAZ10; Fig. 1G). A ring structure was inferred from the FAZ1 and FAZ8 localisation from the spur of fluorescence signal that crossed the flagellum and appeared to be bifurcated or ring-like. This suggested that *L. mexicana* possesses a complex flagellar pocket organisation, including a short linear structure (with additional elaborations) positioned to one side of the flagellum that is homologous to the *T. brucei* FAZ. This region also appeared to include a structure that is homologous to the bilobe (Fig. 1H), similar to the proximal end of the FAZ filament domain densities in *T. brucei* (Esson et al., 2012).

### *L. mexicana* flagellar pocket organisation has many similarities but also key differences to that of *T. brucei*

We characterised the structure of the *L. mexicana* flagellar pocket using electron tomography to generate a 3D reconstruction of the organisation of the basal and pro-basal body, the flagellar pocket, the pocket collar, the flagellum exit from the cell and any structures that could be orthologous to the *T. brucei* FAZ. Analysis was based on tomograms from longitudinal sections of the entire pocket volume of three cells, supported by tomograms from transverse sections through parts of the pocket of a further three cells (to avoid misinterpretation from the lower *z*-resolution in tomograms), at 1–2 nm/voxel (Table S1, Movies 1–5).

**Fig. 2.**
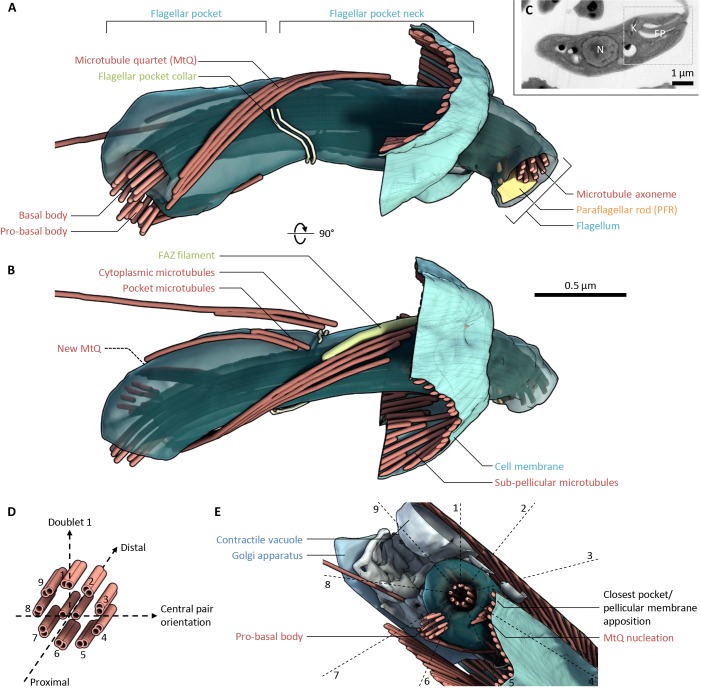
**The organisation of the *Leishmania mexicana* flagellar pocket.** (A) Lateral view of the flagellar pocket structure of a representative promastigote 1K1N *L. mexicana* cell (see C), as determined using serial section electron tomography. Flagellum, flagellar pocket and associated structures are shown, relative to a small portion of the pellicular microtubules and membrane. Generated using tomogram PL1*.* (B) The structure shown in A rotated 90° around the horizontal axis. (C) Low-magnification electron micrograph of the cell whose flagellar pocket is shown in A and B. Section 4 of the six used to build the tomogram is shown. The location of the nucleus (N), kinetoplast (K), flagellar pocket (FP) and region reconstructed using electron tomography (box) are indicated. (D) The standard axoneme doublet microtubule numbering scheme and corresponding coordinate system that we used to describe organelle locations relative to the flagellum within the flagellar pocket. (E) Anterior to posterior view of the base of the flagellar pocket shown in A and B. Five structures and/or organelles, which were found in a consistent position relative to the flagellum and flagellar pocket in all tomograms analysed, are indicated, mapped relative to the axoneme doublet numbering scheme. Generated using tomogram PL1.

The *L. mexicana* flagellar pocket was similar overall to that of *T. brucei* with two distinct domains separated by the flagellar pocket collar, which was visible as a double line of electron-dense material (Fig. 2A,B). The flagellum extended through the pocket with the paraflagellar rod (PFR) present only past the pocket collar. The pocket had an overall curved shape. In the proximal region (before the collar), the pocket was bulbous and surrounded by many small vesicles. In the distal region (after the collar), the pocket narrowed to a cylindrical neck connecting the bulbous proximal pocket to the cell anterior and was surrounded by complex structures with high electron density. A quartet of four microtubules (MtQ) ran as a tight parallel array along the pocket surface. They followed a left-handed helical path, starting near to the basal body, passing through a gap in the collar, then terminating irregularly in the neck region. This irregular termination of the MtQ is unlike that in *T. brucei.* In one tomogram (PL2), it could be seen that the pro-basal body had begun extending into a very short new axoneme, whereas in the remainder, the pro-basal body had not extended. However, in all tomograms analysed, the pro-basal body also had an MtQ, which was much shorter and only extended around half-way to the pocket collar. Note that as *L. mexicana* has few discrete morphological markers of the cell cycle stage in the early cell cycle (Wheeler et al., 2011), we could not determine precisely which cell cycle stage these cells were in, so at which cell cycle stage new MtQ nucleation occurred. Three or four additional microtubules were also always present in two groups – one singlet or pair running along the surface of the pocket and one singlet or pair extending into the cytoplasm. Structures consistent with this organisation were seen in all six promastigote tomograms.

The axoneme central pair of *L. mexicana* has a fixed orientation and can be used to unambiguously number the outer doublets. This provides a polar coordinate system for describing the location of structures in and near the flagellum and pocket using the proximal–distal axis of the axoneme and the angle around the proximal–distal axis, described by using the doublet number (steps of 40° per doublet, clockwise looking proximal to distal) (Fig. 2D).

The flagellar pocket itself was asymmetric and had a consistently asymmetric positioning within the anterior of the cell. Other organelles and structures with a consistently asymmetric position (Fig. 2E) were the pro-basal body (adjacent to doublets 6 and 7 of the axoneme, 200–240°), the nucleation of the MtQ (doublets 4 and 5, 120–160°), the closest apposition of the pocket with one side of the cell pellicle (doublets 3 and 4, 80–120°), the contractile vacuole (doublets 9 and 1, 320–360°) and the Golgi (doublets 7 and 8, 240–280°). These structure and organelle positions were seen in the tomograms of all six promastigote cells where the structure or organelle fell within the tomogram volume.

### FAZ-like structural features are found around the pocket in *L. mexicana*

**Fig. 3.**
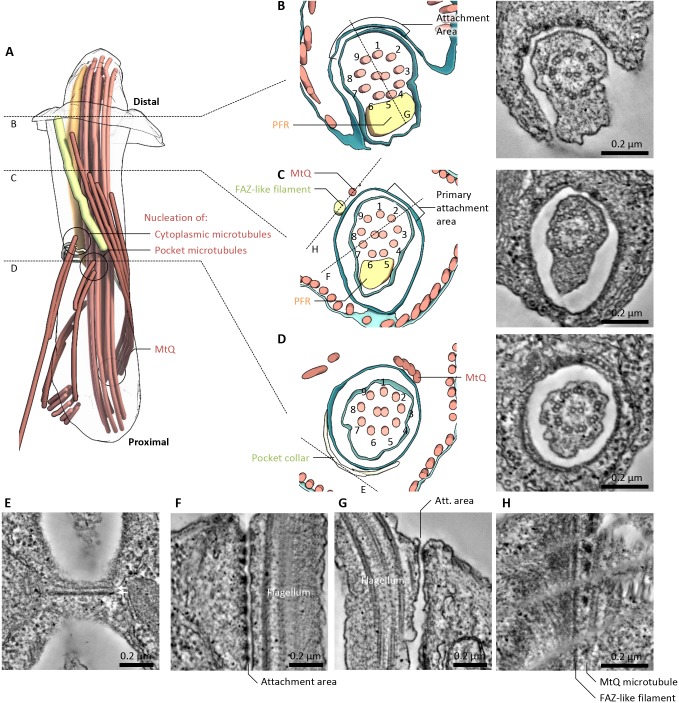
**FAZ-like structures in the *L. mexicana* flagellar pocket neck.** (A) Overview of the key non-membrane structures in the *L. mexicana* flagellar pocket neck. Dashed lines indicate regions of particular interest, illustrated in B–D. Generated using tomogram PL1. (B–D) Transverse views (looking proximal to distal) of the structure of the flagellar pocket neck at three key points along its length, and 10-nm virtual transverse sections (generated from tomogram volumes), showing the electron density corresponding to the structures segmented. (B) The exit of the flagellum from the flagellar pocket. Tomogram PT2. (C) The central portion of the flagellar pocket neck. Tomogram PT1b. (D) The flagellar pocket collar. Tomogram PT1a. Dashed lines indicate the location of longitudinal sections of particular interest, with example images shown in E to H, as indicated. (E-H) 10-nm virtual longitudinal sections (generated from tomogram volumes), illustrating electron densities corresponding to key structures in the flagellar pocket neck region. E and H are tangential to the flagellar membrane, F and G are perpendicular. Oriented with distal upwards. (E) The double line (arrows) of the flagellar pocket collar. Tomogram PL2. (F) The primary flagellum attachment area. Digitally straightened from tomogram PL3. (G) Example of additional flagellum attachment areas (Att. area) at the flagellar pocket neck lip. Tomogram PL1. (H) The electron-dense FAZ filament and neighbouring electron densities, next to the MtQ. Digitally straightened from Tomogram PL2.

The flagellar pocket neck also had a consistent chiral organisation, clearly seen in transverse views of the neck structure (Fig. 3A–D). At the proximal end of the neck, the MtQ passed through an opening in the collar (adjacent to doublets 2 and 3 of the axoneme, 40–80°) with the pocket microtubules starting adjacent to doublets 9 and 1 (320–360°) and the cytoplasmic microtubules starting adjacent to doublet 9 (∼320°) (Fig. 3D). By the mid-section of the neck, the helical organisation of the MtQ had rotated the MtQ position so that it was adjacent to doublets 9 and 1 (320–360°), and the PFR was present next to doublets 5–7 (160–200°) (Fig. 3C). In this region, a regular organisation of the electron density around the neck was visible; there was an electron-dense filament, similar to the *T. brucei* FAZ filament, running parallel to the path of the MtQ (doublets 8 and 9, 280–320°) and there was an area where attachment of the flagellar membrane to the pocket membrane was visible (doublets 1–3, 0–80°) (Fig. 3C). The similarity of the filament and attachment regions to those of *T. brucei* suggests that they are homologous to the structures in the *T. brucei* FAZ. At the distal end of the *Leishmania* neck, the MtQ and FAZ filament had terminated; in this region, the attachment area had extended from spanning adjacent doublets to spanning doublets 8 to 2 (280 to 40°) (Fig. 3B). The cell body protruded further on the side to which the flagellum was attached, giving an asymmetric pocket neck opening (Fig. 3B).

These structural features have distinct appearances in longitudinal sections through the neck region – sections tangential to the pocket membrane at the collar revealed the collar as a double line of electron density, with the more proximal line appearing thicker (Fig. 3E). Sections perpendicular to the pocket membrane in the neck showed the regions of attachment as tight membrane junctions mediated by pseudo-regularly arranged junctional complexes (Fig. 3F,G). Sections at around 40° relative to the axoneme showed junctional complexes along the length of the pocket neck (Fig. 3F), whereas at other orientations, junctional complexes were only ever at the distal limit of the pocket neck (Fig. 3G). The junctional complexes were approximately evenly spaced but were not in a regular array. Sections through the FAZ filament, tangential to the pocket membrane, showed an electron-dense filament that was ∼5 nm wide, which was positioned between a broad region of electron density on the left (looking from the outside of the pocket, with distal oriented up) and the MtQ on the right (Fig. 3H). In distal regions, junctional complexes were present between the FAZ filament and the MtQ (Fig. 3H).

### *T. brucei* FAZ proteins can localise to the *L. mexicana* FAZ

**Fig. 4.**
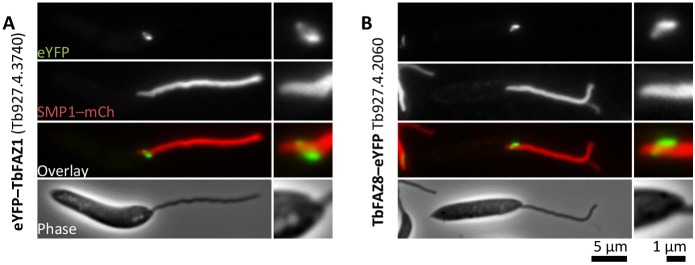
***T. brucei* FAZ proteins localise to the *L. mexicana* flagellar pocket neck.** Fluorescence micrographs of *L. mexicana* promastigotes expressing eYFP fusions (eYFP–) of the indicated *T. brucei* FAZ proteins. SMP1–mCh is a flagellar membrane marker. Detail of the pocket region is shown on the right in each panel. Native eYFP fluorescence shows a localisation similar to that of eYFP fusions of the *L. mexicana* homologs (Fig. 1).

The structures in the *L. mexicana* neck have several similarities to those in the *T. brucei* FAZ – the junctional complexes and the FAZ filament appear similar to the *T. brucei* junctional complexes and FAZ filament, and moreover, both of these structures are associated with the MtQ as in *T. brucei*. Given these similarities, we expressed eYFP fusions of two *T. brucei* FAZ proteins (FAZ1 and FAZ8) in *L. mexicana* to determine whether they could localise to the pocket neck region (Fig. 4). FAZ1 and FAZ8 were selected as the *L. mexicana* homologs of these proteins and showed a localisation pattern (a line with a ring; Fig. 1C,D) that is not observed in *T. brucei*, and the genes were sufficiently small to be readily cloned. Both *T. brucei* FAZ1 and *T. brucei* FAZ8 localised to the distal pocket region of *L. mexicana* and had a similar localisation pattern to that of the eYFP fusions of their respective *L. mexicana* homologs (compare Fig. 4A and Fig. 1C, Fig. 4B and Fig. 1D), supporting the hypothesis that there is a true FAZ in *Leishmania* with discrete homologies to that of *T. brucei*. *Leishmania*-specific localisation occurred for these proteins even though the sequence identity (∼40%, depending on the handling of repetitive regions) between the *T. brucei* and *L. mexicana* homologs was only moderate.

### Pocket organisation is similar in *L. mexicana* promastigotes and amastigotes

*Leishmania* undergoes a large morphological change during the life cycle transition from the promastigote form in the sandfly to the amastigote form inside mammalian macrophages. This involves a restructuring of the flagellum from a motile 9+2 axoneme to a collapsed 9+0 (9v) organisation (Gluenz et al., 2010). We used electron tomography to assess how the flagellar pocket and *Leishmania* FAZ structures are modified in this different life cycle stage. Analysis was based on tomograms from longitudinal sections of the pocket region of three amastigote cells 96 h after infection of J774 macrophages at 1–3 nm/voxel (Table S1, Movies 6–8).

**Fig. 5.**
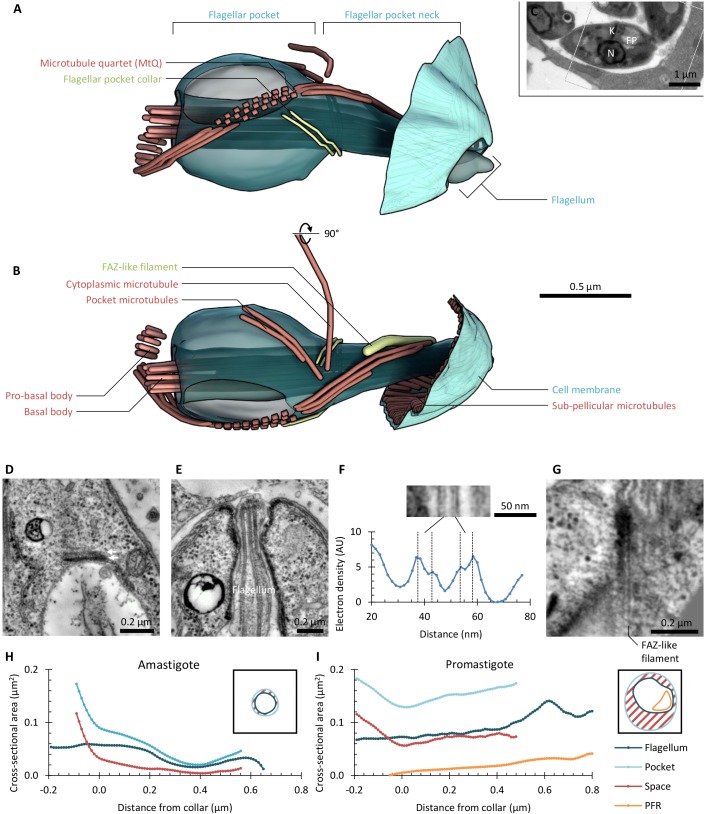
**Adaptation of the *L. mexicana* flagellar pocket and FAZ structures in the amastigote.** (A) Lateral view of the flagellar pocket structure of a representative amastigote 1K1N *L. mexicana* cell (see C), as determined using serial section electron tomography. Flagellum, flagellar pocket and associated structures are shown, relative to a small portion of the pellicular microtubules and membrane. The inferred path of the MtQ out of the tomogram volume is indicated with dashed structures. Generated from Tomogram AL2. (B) The structure shown in A rotated 90° around the horizontal axis. (C) Low-magnification electron micrograph of the cell whose flagellar pocket is shown in A and B. Section 1 of the three used to build the tomogram is shown. The location of the nucleus (N), kinetoplast (K), flagellar pocket (FP) and region reconstructed by using electron tomography (box) are indicated. (D–G) 10-nm virtual longitudinal sections (generated from tomogram volumes), illustrating electron densities corresponding to key structures in the flagellar pocket neck region. D and G are tangential to the flagellar membrane, E and F are perpendicular. Oriented with distal upwards. (D) The double line of the flagellar pocket collar (arrows). Tomogram AL2. (E) The flagellum embedded in the attachment area. Tomogram PL2. (F) Detail of the typical flagellum and flagellar pocket membrane spacing in the attachment area, and quantitation of the corresponding electron density profile. Both lipid bilayers can be seen, separated by ∼10 nm. Generated using tomogram PL1. (G) The electron-dense FAZ filament and neighbouring electron densities. Digitally straightened from tomogram AL3. (H,I) Cross-sectional area of the flagellum and flagellar pocket, and remaining space allowing entry to the flagellar pocket, for a representative amastigote and promastigote. The small cartoon cross-sections illustrate the areas measured. Generated using tomograms PL1 and AL2, respectively.

The pocket organisation in the amastigote was similar overall to that in the promastigote (Fig. 5), with the exception of the narrower flagellum neck region (as the flagellum axoneme has a 9v structure with no PFR) and a larger pocket width (Fig. 5A,B). The flagellar pocket collar, MtQ, pocket and cytoplasmic microtubules, and FAZ filament were all present (Fig. 5A,B), although the microtubule organisation was more variable than in the promastigote. Two cells lacked one of the microtubules of the MtQ for much of the MtQ length, one cell lacked any cytoplasmic microtubules and one had an extension of a pocket microtubule in the distal direction. All cells also lacked a MtQ associated with the pro-basal body that had been seen in promastigotes.

In the absence of a central pair, axoneme orientation was determined assuming that the pro-basal body was positioned between doublets 6 and 7 (200–240°), as in the promastigote (Fig. 2E). This indicated that the position of MtQ nucleation, closest flagellar pocket and pellicular membrane apposition, transit of the MtQ through the pocket collar, and nucleation of the pocket and cytoplasmic microtubules occurred at the same location as in the promastigote. In the distal pocket neck, the irregular inward collapse of the outer doublets makes axoneme orientation a meaningless measure; however, the FAZ filament was positioned parallel to the trajectory of the MtQ (Fig. 5A,B,G). Owing to a lack of a PFR, the tip of the flagellum at the exit of the flagellar pocket was more symmetrical than the promastigote. The amastigote flagellar pocket exit also did not have such an asymmetric extension of the cell body (Fig. 5A,B).

### Flagellum attachment area organisation has adaptations in *L. mexicana* amastigotes

Unlike in the promastigote, clearly separated junctional complexes were not visible, instead, large areas showed tight flagellar and pocket membrane links with close apposition of the membrane bilayers in many areas (Fig. 5E,F). As a result, the space between the flagellar and pocket membranes for access to the flagellar pocket appears to be greatly reduced. We quantified the cross-sectional area of the flagellum, the flagellar pocket and the resulting opening at 10-nm steps proximal to distal, from 100 nm before the flagellar pocket collar to beyond the distal end of the pocket, for the most complete promastigote and amastigote tomograms (PL1 and AL2) from the segmented membrane models (Fig. 5H,I). The promastigote and amastigote had comparable flagellum and pocket cross-sectional areas at the collar (around 0.05 and 0.12 μm^2^, respectively); however, the promastigote pocket neck was narrowest at the collar – the pocket, flagellum and cross-sectional area of the access space all increased with distance from the collar. In contrast, the amastigote pocket, flagellum and access space all had a constriction in the cross-sectional area distal of the collar by 0.4 μm, with the cross-section of the area through which material can access the flagellar pocket dropping to under 0.01 μm^2^ (assuming no blocking of access by the adhesion structures themselves).

**Fig. 6.**
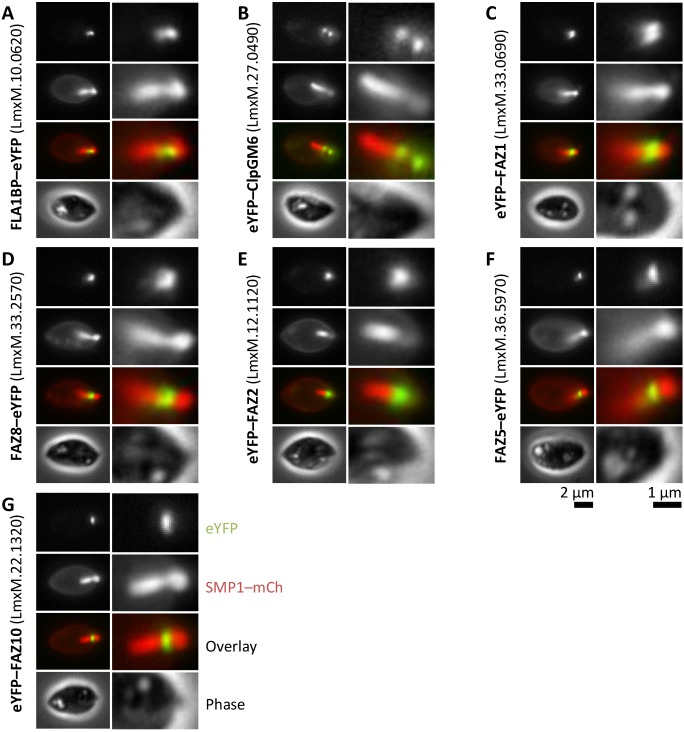
***L. mexicana* FAZ protein localisations in the amastigote.** Fluorescence micrographs of axenic *L. mexicana* amastigotes expressing eYFP fusions (eYFP–) of the indicated *L. mexicana* homologs of *T. brucei* FAZ proteins. SMP1–mCh is a flagellar membrane marker. Detail of the pocket region is shown on the right in each panel. Native eYFP fluorescence shows a change in structure from that in the promastigote.

The differences in structure of the promastigote and amastigote pocket and flagellum attachment areas suggest that the localisation of *L. mexicana* FAZ protein homologs should show changes in the amastigote. We analysed this by triggering differentiation in our cell lines that expressed eYFP fusions of FAZ1, FAZ2, FAZ5, FAZ8, FAZ10, ClpGM6 and FLA1BP and the SMP1–mCh flagellum membrane marker into axenic amastigotes (Fig. 6). The constriction in flagellum width in the pocket neck was clearly visible in the SMP1–mCh signal. The FAZ proteins could be separated into three classes based on the localisation patterns – a line or ring in the cytoplasm across the flagellar neck constriction (FAZ1, FAZ2, FAZ5, FAZ10, FAZ8), in the flagellum in the neck constriction following the shape of the flagellum (FLA1BP) or in the flagellum proximal and distal of the neck constriction (ClpGM6). This is consistent with the restructuring of the extended attachment region in the promastigote pocket neck, with distinct neck and lip attachment regions, into a compacted structure focused on the neck constriction in the amastigote, as we saw using electron tomography (Figs 2, 3 and 5). It also suggests a function of ClpGM6 outside of the primary attachment areas and serves to further highlight the diversity of the overall FAZ structures that these FAZ proteins can form.

## DISCUSSION

Here, we provide an integrated view of the *Leishmania* flagellar pocket region in both the promastigote and amastigote using electron tomography analysis and eYFP tagging. Our data suggest that the *Leishmania* flagellar pocket and associated FAZ structure (which includes a short FAZ filament) have crucial roles in morphogenesis, signalling and motility, like their *T. brucei* counterparts. The flagellar pocket in *T. brucei* is essential for pathogenicity; it is the only site for exo- and endocytosis and also contains numerous receptors that are crucial for immune evasion and survival (Engstler et al., 2007; Field and Carrington, 2009). In *Leishmania*, the flagellar pocket is likely to be equally important, especially in the intracellular amastigote form, as molecules that influence the behaviour of both the parasite and the macrophage might transit through it.

### Flagellar pocket and FAZ structures in *L. mexicana* and *T. brucei*

Overall, the flagellar pocket structure in *L. mexicana* is strikingly similar to that previously described for *T. brucei* (Lacomble et al., 2009, 2010). Both share the same core structures (MtQ and collar) in a similar asymmetric organisation, with this similarity including the arrangement of the pro-basal body and Golgi around the pocket. Surprisingly, the similarities also extend into the flagellar pocket neck with the anchoring of the *L. mexicana* flagellum in the neck region, showing similarity to the proximal end of the *T. brucei* FAZ, particularly in the promastigote. Furthermore, *L. mexicana* FAZ protein homologs localised to this neck region, and *T. brucei* FAZ proteins expressed in *L. mexicana* had the same asymmetry of localisation as their *L. mexicana* homologs. The attachments we and others have described in the *L. mexicana* pocket neck region therefore appear to be truly homologous to the *T. brucei* FAZ – *Leishmania* have a FAZ, despite not having a trypomastigote morphology with a laterally attached flagellum.

A naïve assumption from the overall promastigote cell shape is that *Leishmania* are bilaterally or radially symmetric. The chiral organisation of the pocket and presence of an asymmetric FAZ shows neither is the case, with the flagellar pocket consistently positioned asymmetrically within the cell anterior and the plane of the flagellar beat (perpendicular to the axoneme central pair) not aligned with the FAZ filament and attachment zone. This suggests that a possible role of the *Leishmania* FAZ is in defining radial cell polarity, with the orientation of the cell and cytoplasmic structures rigidly defined by the orientation of the axoneme and flagellar beat plane through the FAZ, or vice versa*.* This might have important consequences for cell swimming behaviours through subtle asymmetries in cell shape.

There were some small differences in pocket organisation. The pocket collar in *L. mexicana* was a clear double filament around the pocket, whereas in *T. brucei*, it was a single filament, perhaps indicating an additional collar protein and/or structure in *L. mexicana*. In promastigotes, we always saw the pro-basal body with an associated short MtQ. In contrast, we did not see a pro-basal body-associated MtQ in any of the amastigote tomograms, perhaps suggestive of a more quiescent state or longer cell cycle. Finally, *L. mexicana* had additional pocket and cytoplasmic microtubules nucleated near the collar, of which one is likely to be the lysosome-associated microtubule previously described (Weise et al., 2000).

The differences in FAZ organisation are more extensive. In *T. brucei* the FAZ structures are arranged in an extended linear structure, with a filament and a line of regularly spaced junctional complexes (Höög et al., 2012; Sherwin and Gull, 1989; Vickerman, 1969). Filamentous structures were seen in both promastigote and amastigote *L. mexicana*, yet junctional complexes did not follow the regular linear organisation seen in *T. brucei*, especially in the amastigote. In *T. brucei*, the junctional complexes consistently link to doublet 7 of the axoneme (Vickerman, 1969); however, in *L. mexicana* promastigotes, the most commonly used doublets were 1, 2 and 3, and in amastigotes, attachment extended around the entire flagellum. Therefore, although the *L. mexicana* FAZ structure is clearly homologous to the *T. brucei* FAZ and is made up of homologous proteins, there are significant structural adaptations.

Despite the differences in FAZ organisation between *T. brucei* and *L. mexicana*, the structures share a key organisational similarity – the FAZ runs from the point in the cell at which the division cleavage furrow starts to the flagellar pocket collar (Robinson et al., 1995; Wheeler et al., 2011, 2013a). This suggests a possible role of the *Leishmania* FAZ in the propagation of cell structure information for division, as in *T. brucei*, ensuring that single-copy organelles orientated relative to the MtQ and axoneme are positioned correctly for segregation into daughter cells by the spatial constraints conferred by the FAZ and its links to the sub-pellicular microtubules. If the ancestral morphology of the trypanosomatids was the promastigote, then this plausibly could have been the ancestral FAZ function.

### Insights into FAZ protein biology

**Fig. 7.**
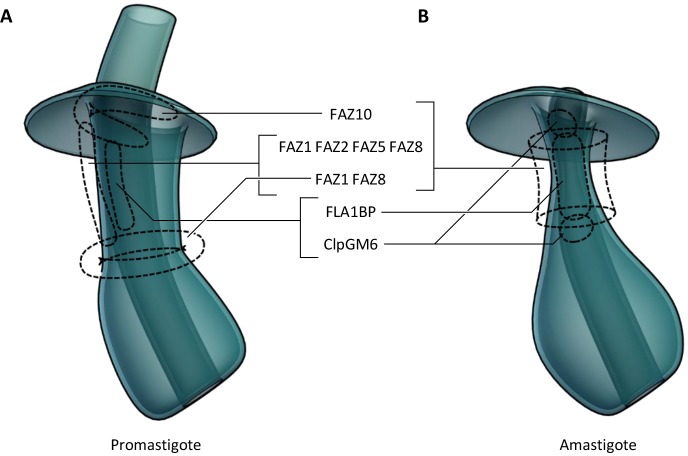
**Possible FAZ protein locations in the pocket ultrastructure.** 3D cartoon of possible protein localisations, inferred from light micrographs of eYFP fusions of FAZ proteins and the pocket structure derived from electron tomography analysis. (A) The promastigote flagellar pocket. (B) The amastigote flagellar pocket.

In *T. brucei*, the FAZ is essentially a linear structure, whereas the FAZ in *L. mexicana* is a less linear structure with spatial separation between the junctional complexes and the FAZ filament, and moreover, the *L. mexicana* FAZ shows differences in structure between the two life cycle stages as a result of adaptation. Although we have no direct evidence for the ultrastructural localisation of different FAZ proteins, we can infer plausible localisations from the combination of light and electron microscopy (Fig. 7). The different categories of FAZ protein localisation suggest that the FAZ proteins can be split into different cohorts that together form higher-order structures, which we term component modules, that together with other component modules are responsible for determining the overall shape of the pocket region. *L. mexicana* therefore provides new opportunities to infer FAZ protein function.

FAZ1, FAZ2, FAZ5 and FAZ8 could form a functional group because they have a set of similar localisations both in the promastigote and amastigote, and it is possible that they have a role in forming the filament or the surrounding electron density on the cytoplasmic side of the FAZ. In addition, both FAZ1 and FAZ8 form a potential ring structure that might coincide with the flagellar pocket collar, and therefore, these proteins could act to link the FAZ filament to the collar. FAZ5 (unlike FAZ1, FAZ2 or FAZ8) contains multiple predicted transmembrane domains, so might have a function in linking the FAZ filament to the membrane.

FLA1BP and ClpGM6 are flagellum-side components in *T. brucei* (Hayes et al., 2014; Sun et al., 2013), and the localisation we observed is consistent with the same being true in *Leishmania*. FLA1BP is clearly associated with regions of attachment (linear structure in the promastigote and at the neck constriction in the amastigote). The exclusion of ClpGM6 from neck constriction in the amastigote suggests a function that does not primarily occur in attachment but that occurs elsewhere in the FAZ structure.

FAZ10 seems to have a distinct function – in the promastigote, it is associated with the large region of attachment located at the exit from the flagellar pocket, and in the amastigote, it is associated with the extensive attachment around the pocket neck constriction, suggesting involvement with these wider areas of attachment. In *T. brucei* bloodstream forms, the monoclonal antibody DOT1 (Woods et al., 1989) epitope has a similar behaviour – it is found in a large region of attachment around the tip of the new growing flagellum (Hughes et al., 2013), so DOT1 might detect *T. brucei* FAZ10 or another FAZ protein in this functional group.

None of the FAZ proteins examined here (FAZ1, FAZ2, FAZ5, FAZ8, FLA1BP, ClpGM6, FAZ10) seem entirely responsible for attachment. There are two regions of attachment in promastigote *L. mexicana* – the linear structure region through the neck and the region around the exit point from the neck. None of these proteins seemed to localise to both of these regions; therefore, there are either different attachment structures in different regions or as yet unidentified proteins that form the actual adhesion structures. However, this conclusion is based on the localisation of eYFP fusions of these structural proteins, and of course, always has a caveat of the wild-type protein having a different localisation.

As mentioned above, these FAZ proteins show flexibility in the structures they can form. For example, FAZ10 can form a linear structure in *T. brucei*, whereas the *L. mexicana* homolog can form a horseshoe or ring around the flagellum pocket neck. Furthermore, *T. brucei* FAZ1 and FAZ8 form a linear structure in *T. brucei* but, when expressed in *L. mexicana*, have the capacity to form more complex structures, including what appears to be a ring around the flagellum. However, we do not know if they are able to functionally replace the *L. mexicana* homologs.

### Implications for mechanisms of trypanosomatid parasite morphogenesis

Trypanosomatids have a set of characteristic morphologies, in part defined by the positioning of the flagellar pocket and whether or not they have a laterally attached flagellum (Hoare and Wallace, 1966). Taking the promastigote morphology as the ancestral morphology (Flegontov et al., 2013), the structure of the *L. mexicana* FAZ can be used to predict what the ultrastructure of these morphologies is likely to be, what their molecular composition is and how they could have evolved. Firstly, the opisthomastigote appears to simply have an extension of the neck region, pushing the collar and pocket deeper into the cell, based on the presence of the PFR in the extended pocket (Janovy et al., 1974; Rowton et al., 1981; Yoshida et al., 1978). Secondly, the choanomastigote appears to have an elaboration of the attachment around the distal end of the flagellar pocket neck, the region characterised by the presence of FAZ10, into a larger attachment structure previously described as containing many desmosome-like junctional complexes (Brooker, 1970, 1971; Brooks, 1978; Kusel et al., 1967; Soares et al., 1986). Thirdly, the laterally attached flagellum of the trypomastigote or epimastigote appears to require shifting of the flagellar pocket to anchor within the sub-pellicular array (rather than at an opening at the end of the array), the extension of the MtQ and FAZ filament into the sub-pellicular array, and relocation of junctional complexes into the vicinity of the FAZ filament. It will be of interest to determine how these morphological changes have co-evolved with the morphogenesis of cell shape through the cell cycle, which differs between *Leishmania* (Ambit et al., 2011; Wheeler et al., 2011) and trypanosomes (Elias et al., 2007; Robinson et al., 1995; Sherwin and Gull, 1989; Wheeler et al., 2013a).

The difference in organisation of the promastigote and amastigote pocket neck and FAZ region implies that the amastigote morphology represents an adaptation that minimises total surface area. The amastigote is small and near spherical, suggesting a minimisation of cell volume (metabolic load for growth and maintenance) and/or surface area (in the potentially harmful environment of the macrophage endocytic system). Access to the flagellar pocket in the promastigote is relatively easy, with a large cross-sectional area open to fluid access, whereas in the amastigote, the flagellum essentially plugs the pocket entrance, acting to further reduce the exposed surface area of the parasite. In the promastigote, the structure of the narrowest part of the neck appears to be modulated by the flagellar pocket collar, whereas in the amastigote, it is a region dominated by the FAZ structures. The FAZ therefore appears to be involved in regulating access to the flagellar pocket. In *T. brucei*, the MtQ has been implicated in keeping an access channel to the flagellar pocket open (Gadelha et al., 2009), and *T. brucei* MORN1 has recently been identified as controlling access to the flagellar pocket (Morriswood and Schmidt, 2015). *T. brucei* MORN1 is associated with the pocket neck and the proximal end of the *T. brucei* FAZ (Esson et al., 2012), and it seems likely that FAZ structure could be modulating *L. mexicana* MORN1 control of pocket access in *L. mexicana.* Not only do *Leishmania* have a FAZ despite lacking a laterally attached flagellum, but this FAZ plausibly has a key function in adapting morphology throughout the life cycle in order to modify the pocket structure.

This discovery and description of the *Leishmania* FAZ in the context of the flagellar pocket architecture has relevance to fundamental issues of how possession, expression and the regulation of assembly of component modules change throughout the life cycle and evolution to orchestrate different cell shapes and forms.

## MATERIALS AND METHODS

All reagents were purchased from Sigma-Aldrich unless stated.

### *L. mexicana* culture

*L. mexicana* (World Health Organization strain MNYC/BZ/62/M397) promastigotes were grown in M199 medium with Earle's salts and L-glutamine (Thermo Fisher), supplemented with 10% foetal calf serum (FCS) (Thermo Fisher), 5 mM HEPES·NaOH (pH 7.4), 26 mM NaHCO_3_ and 5 µg/ml haemin, at 28°C. Cells were maintained in logarithmic growth at culture densities between 1×10^5^ cells/ml and 1×10^7^ cells/ml through regular subculture (Wheeler et al., 2011). Culture densities were measured with a CASY model TT cell counter (Roche Diagnostics). Axenic amastigotes were generated by subculturing into Schneider's *Drosophila* medium (Thermo Fisher) supplemented with 20% FCS (Thermo Fisher) and 25 mM MES·HCl (pH 5.5) at 34°C under 5% CO_2_ (Bates, 1994), and growth for 72 h without subculture. Amastigotes were generated by infection of J774 macrophages with stationary-phase promastigotes and allowed to differentiate into amastigotes for 72 h. J774 macrophages were grown in RPMI (Thermo Fisher), supplemented with 10% FCS (Thermo Fisher), at 37°C (prior to infection) or at 34°C (after infection) under 5% CO_2_.

### Tagging of proteins

For the eYFP tagging of the *L. mexicana* proteins, the corresponding ORFs and UTRs were cloned into the pLEnTv2-YB plasmid, as previously described (Dean et al., 2015). To tag FAZ1, FAZ2, ClpGM6, FAZ10 and LRRP1 at the N-terminus with eYFP, ∼500 bp of the 5′ end of the gene directly after the start codon and ∼500 bp of 5′ UTR directly upstream of the start codon was amplified from genomic DNA. An *Xba*I site was added to the forward primer and a *Not*I site added to the reverse primer for the amplification of the ORF. A *Not*I site was added to the forward primer and a *Bam*HI site added to the reverse primer for the amplification of the 5′ UTR. The resulting PCR fragments were digested with the appropriate restriction enzymes and cloned into the pLEnTv2-YB plasmid that had been digested with *Xba*I and *Bam*HI (New England Biolabs). To tag FLA1BP, FAZ8 and FAZ5 at the C-terminus with eYFP, ∼500 bp of the 3′ end of the gene directly before the stop codon and ∼500 bp of 3′ UTR directly downstream of the stop codon was amplified from genomic DNA. A *Not*I site was added to the forward primer and a *Spe*I site added to the reverse primer for the amplification of the ORF. A *Hind*III site was added to the forward primer and a *Not*I site added to the reverse primer for the amplification of the 3′ UTR. The resulting PCR fragments were digested with the appropriate restriction enzymes and cloned into the pLEnTv2-YB plasmid that had been digested with *Spe*I and *Hind*III (New England Biolabs). The resulting plasmids were linearised with *Not*I (NEB, Hitchin, UK) and then ethanol precipitated before transfection.

For Myc-tagging of the *L. mexicana* BILBO1 homolog, a fusion PCR approach was taken, as previously described (Dean et al., 2015). To tag BILBO1 at the N-terminus with a Myc tag, ∼500 bp of the 5′ end of the gene directly after the start codon and ∼500 bp of 5′ UTR directly upstream of the start codon was amplified from genomic DNA. The forward primer for the amplification of the BILBO1 ORF had a 27-bp region of homology to the 3′ end of the actin 5′ UTR at its 5′ end followed by a Myc-epitope tag that was in-frame with the ORF. The reverse primer for the amplification of the 5′ UTR had a 25-bp region of identity to the start of the blasticidin-resistance gene. Three pieces of DNA, the 500 bp UTR and ORF fragments amplified from genomic DNA, and the region from the pPOTv2 plasmid containing the blasticidin-resistance gene followed by the *aldolase* 3′ UTR and then the *actin* 5′ UTR, released using an *Eco*RI *Hind*III (New England Biolabs) digest were combined in a fusion PCR. The PCR required 30 amplification cycles, five of which were without any primers, followed by 25 cycles using nested primers that annealed 40 bp from the 5′ end of the UTR fragment and 40 bp from the 3′ end of the gene fragment. After amplification, the construct was purified and then used for the transfection.

### Expression of the *T. brucei* FAZ proteins in *L. mexicana*

For the expression of the *T. brucei* FAZ proteins in *L. mexicana*, a new modular constitutive expression plasmid was made. The plasmid integrates into the β-tubulin array. From the 5′ end of the plasmid, there is the tubulin upstream targeting sequence followed by the *Crithidia fasciculata PGKB* 5′ UTR, followed by a Ty–eYFP–Ty ORF (where Ty is an epitope from the *Saccharomyces Cerevisiae* Ty1 virus-like particle) and then the *C. fasciculata PGKA* 3′ UTR and *PGKB* 5′ UTR, next is the blasticidin ORF and then the *C. fasciculata GSS* 3′ UTR and finally the tubulin downstream targeting sequence. Every ORF and UTR can be readily exchanged as each component is flanked by unique restriction enzymes. The plasmid can support the expression of both N- and C-terminally eYFP-tagged proteins. To express *T. brucei* FAZ1 with an N-terminal eYFP tag, the *T. brucei* FAZ1 ORF was cloned into the *Xba*I *BamH*I sites, and for expression of *T. brucei* FAZ8 with a C-terminal eYFP tag, the *T. brucei* FAZ8 ORF was cloned into the *Hind*III *Spe*I sites. The plasmids were linearised with *Acc65*I and *Bgl*II (New England Biolabs) and ethanol precipitated before transfection. The plasmids and fusion PCR construct were electroporated, as previously described (Dean et al., 2015), using program X-001 on a Nucleofector 2b instrument (Lonza).

### Immunofluorescence analysis and western blotting

All the cell lines expressing eYFP tagged proteins were examined by using live-cell microscopy. The cells were washed three times in PBS, resuspended in PBS with Hoescht 33342 (1 µg/ml) and then 10 μl placed on a poly-lysine slide. The cells were imaged using a DM5500B (Leica, Milton Keynes, UK) microscope controlled by the Micromanager software with 100×/1.4 objective and Neo 5.5 sCMOS (Andor, Belfast, UK) camera (Edelstein et al., 2010). Immunofluorescence analysis of the Myc–BILBO1 cell line was performed by washing the cells three times in PBS, resuspending the cells in PBS and allowing them to settle on a poly-lysine slide. The cells were fixed with 4% (v/v) formaldehyde for 10 min, and the excess formaldehyde was quenched by washing the slides in PBS with 1% (w/v) glycine for 5 min. The cells were blocked for 1 h with blocking buffer [PBS with 1% (w/v) BSA]. After blocking, the cells were incubated with undiluted anti-Myc antibody clone 9E10 (Evan et al., 1985) for 1 h and then washed thoroughly with PBS before incubating with 1:200 diluted FITC-conjugated rabbit anti-mouse IgG antibody (catalogue number F0261, DAKO) in blocking buffer. The slides were thoroughly washed before mounting with DABCO containing DAPI (100 ng/ml). The cells were imaged using a Leica DM5500B microscope.

Expression of eYFP and Myc fusion proteins were confirmed by western blotting. Cell lysates were resolved on SDS-PAGE gels, transferred to nitrocellulose membrane and probed with 1:2000 diluted rabbit anti-GFP (catalogue number A11122, Thermo Fisher), then 1:5000 diluted horseradish peroxidase (HRP)-conjugated goat anti-rabbit (catalogue number P0448, Dako, Ely, UK) or 1:10 diluted clone 9E10 then 1:20,000 HRP-conjugated rabbit anti-mouse IgG (catalogue number A9044, Sigma-Aldrich), and the signals were detected by using enhanced chemiluminescence.

### Electron tomography imaging

Promastigotes and amastigotes in J774 macrophages were prepared for microscopy as previously described (Gluenz et al., 2015; Höög et al., 2010). Briefly, cells were fixed in culture with 2.5% (v/v) glutaraldehyde (TAAB, Aldermaston, UK) for 5 min, harvested by centrifugation (for promastigotes) or by scraping and then centrifugation (for amastigotes in macrophages) then washed and resuspended in 200 mM phosphate buffer (pH 7.0) with 2.5% glutaraldehyde and 2.0% paraformaldehyde for 2 h. The pellet was washed, post-fixed with 1% osmium tetroxide (Amsbio, Abingdon, UK) for 2 h and stained *en bloc* with 2% uranyl acetate (Amsbio, Abingdon, UK) for 2 h, then dehydrated in an ethanol series and embedded in Agar 100 resin (Agar Scientific, Stansted, UK). Serial sections with a nominal thickness of between 150 and 200 nm (for 100 kV microscopes) or between 250 nm and 400 nm (for 300 kV microscopes) were cut and collected on formvar-coated slot grids, and stained with Reynolds lead citrate (TAAB, Aldermaston, UK) for 2 min.

Tilt series of images were captured on a Tecnai 12 or Tecnai T30 instrument with an Ultrascan 1000 CCD camera between −55° and 55°, and between −64° and 64°, respectively, using SerialEM software (Mastronarde, 2003). The tilt series were aligned using fiducial-less patch tracking, and tomographic volumes were generated by back projection. The tomograms of serial sections were then joined to generate the complete tomogram volume. Tomogram building and joining was performed using eTomo, part IMOD (Kremer et al., 1996; Mastronarde, 1997). Structures in the tomograms were manually traced or segmented using 3DMOD, also part of IMOD (Kremer et al., 1996), then refined and rendered for display using Blender (http://www.blender.org). Virtual sections through the tomogram volumes were generated using ImageJ (Collins, 2007). Summary videos showing the tomogram volume and segmentation (Supplementary Movies 1-8) are hosted on FigShare (https://dx.doi.org/10.6084/m9.figshare.1595927.v1).
